# ‘Double-ring’–like dysplastic mitral leaflet associated with atrioventricular septal defect mimicking endocarditis: computed tomography as key to diagnosis

**DOI:** 10.1093/ehjcr/ytag007

**Published:** 2026-01-09

**Authors:** Pietro G Lacaita, Silvana Müller, Johannes Deeg, Gudrun M Feuchtner

**Affiliations:** Department of Radiology, Medical University Innsbruck, Anichstrasse 35, Innsbruck A-6020, Austria; Department of Internal Medicine III, Cardiology, Medical University Innsbruck, Austria; Department of Radiology, Medical University Innsbruck, Anichstrasse 35, Innsbruck A-6020, Austria; Department of Radiology, Medical University Innsbruck, Anichstrasse 35, Innsbruck A-6020, Austria

## Case description

**Figure 1:**
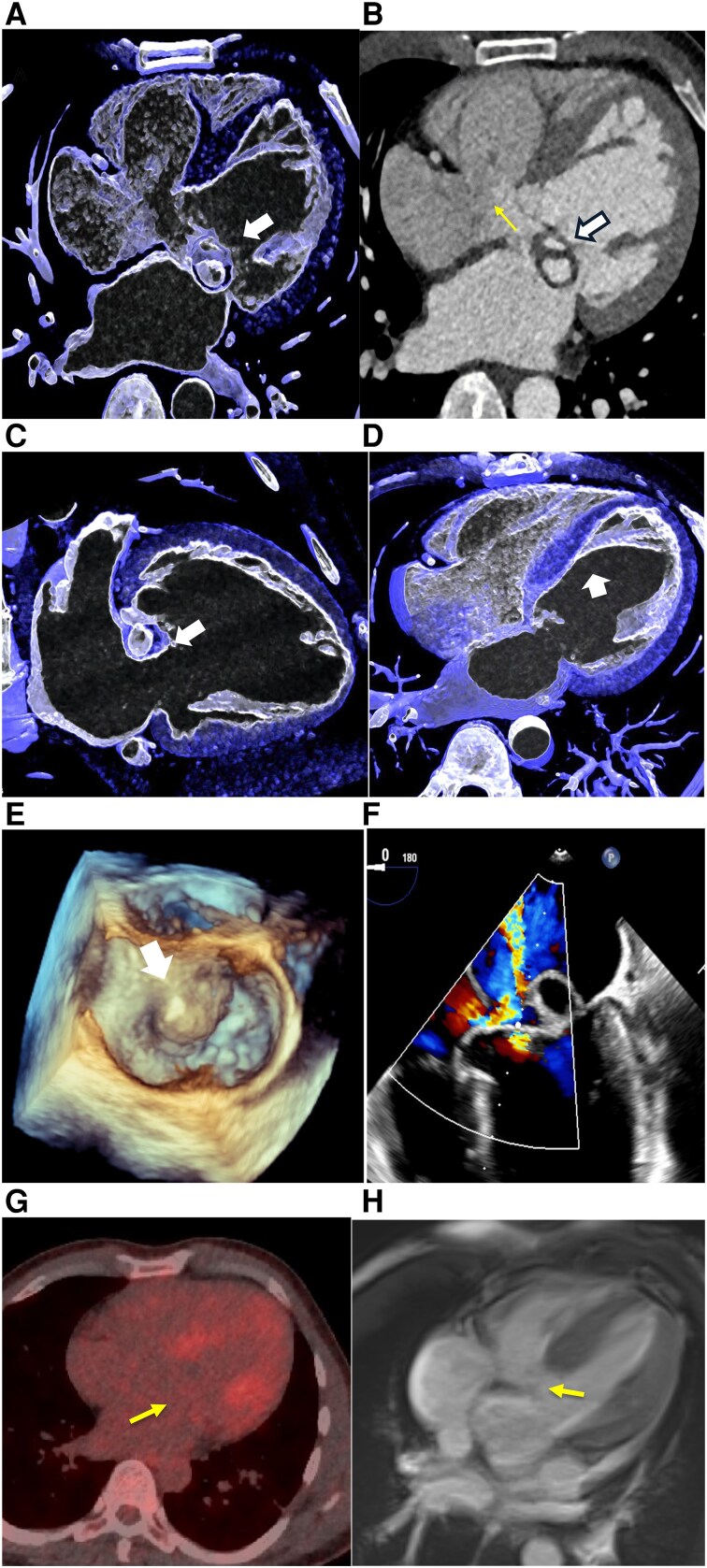
Multi-modality imaging demonstrates the diagnostic features of an atypical mitral leaflet appearing as ‘double-ring’ on coronary computed tomography, which was associated with a previously undiagnosed atrioventricular (AV) canal defect. (*A*) Coronary computed tomography: A short-axis view highlights the septal defect with the dysplastic anterior mitral leaflet and a ‘double-ring’ appearance (white thick arrow). The incomplete atrial and membranous ventricular septum is clearly visible (AV canal) (yellow thin arrow). (*B*) Axial multi-planar reconstruction shows the ‘double-ring’ anterior leaflet and provides detailed visualization of the AV canal defect. (*C*) Ring-like lesion (two-chamber view) attached to the anterior mitral leaflet. (*D*) Atypical insertion of the anterior papillary muscle at the mid-ventricular septum. (*E*) Three-dimensional transoesophageal echocardiography illustrates the dysplastic prolapsing anterior mitral leaflet. (*F*) Doppler transoesophageal echocardiography shows a shunt at the AV level and the dysplastic anterior mitral leaflet and prolapse (see Supplementary material online, *Movie S4*). (*G*) ^18^FDG-PET (left) showed no suspicious tracer uptake, and (*H*) cardiac magnetic resonance imaging confirmed partial AV canal. T1-mapping (not shown) revealed early diffuse myocardial fibrosis with preserved LVEF 56% and confirmed atrioventricular septal defect.

A 41-year-old-male presented with back pain, elevated C-reactive protein (CRP, 9.81 mg/dL), and a systolic murmur. Two blood cultures were positive (*Streptococcus epidermidis*). Transoesophageal echocardiography (TEE) showed an atypical vegetation-like mass with destruction of the anterior mitral leaflet (AML) and severe mitral regurgitation. Oral penicillin therapy was initiated. Twelve days later, he re-presented with persistent back pain (CRP: 2.41 mg/dL). Three blood cultures were negative. Endocarditis remained as ‘possible’ based on the Duke criteria. Cardiac surgery was considered. Coronary computed tomography (CTA) showed an atypical ‘double-ring’–like configuration of the AML and an atrioventricular septal defect (*Figure 1A–C*). The anterior papillary muscle was abnormally inserted to the mid-ventricular septum (*Figure 1D*). Diagnosis of dysplastic AML with prolapse was confirmed by TEE (*Figure 1E* and *F*). Supplemental movies show CTA (1–3) and TEE (4–5) findings. Positron emission tomography (PET) imaging showed no ^18^fluorodeoxyglucose-positron emission tomography (FDG-PET)-tracer uptake (*Figure 1G*). Right heart catheterization demonstrated mild pulmonary hypertension (left pulmonary artery: 53/21/30 mmHg). The patient was scheduled for elective cardiac surgery. During 3 months of follow-up, he was asymptomatic and ultimately declined surgery. Differential diagnosis: A prior underlying inflammatory process superimposed on a dysplastic AML may have caused the atypical formation of a ‘double-ring’ mimicking a vegetation and represents a diagnostic pitfall. Other lesions that can mimic vegetations are tumours.^1^ Atrioventricular septal defect can be further associated with a ‘cleft’.^2^

Our case highlights the pivotal role of multi-modality imaging with TEE, CTA, and ^18^FDG-PET in patients with possible endocarditis, where cardiac computed tomography is a Class IB indication according to the European Society of Cardiology 2023 guidelines,^3^ particularly when valvular lesions are complex or atypical. Atypical lesions such as the ‘double-ring’ sign represent a diagnostic pitfall in differentiating congenital anomalies from vegetations.

## Supplementary Material

ytag007_Supplementary_Data

## Data Availability

The data underlying this article are available in the article and in its online Supplementary material.

## Supplementary material

**Consent:** Patient consent was obtained—in compliance with COPE guidelines.
